# Antiretroviral Drug Discovery Targeting the HIV-1 Nef Virulence Factor

**DOI:** 10.3390/v14092025

**Published:** 2022-09-13

**Authors:** Lori A. Emert-Sedlak, Haibin Shi, Colin M. Tice, Li Chen, John J. Alvarado, Sherry T. Shu, Shoucheng Du, Catherine E. Thomas, Jay E. Wrobel, Allen B. Reitz, Thomas E. Smithgall

**Affiliations:** 1 Department of Microbiology and Molecular Genetics, University of Pittsburgh School of Medicine, Pittsburgh, PA 15219, USA; 2 Fox Chase Chemical Diversity Center, Inc., Pennsylvania Biotechnology Center, Doylestown, PA 18902, USA

**Keywords:** HIV-1, AIDS, Nef, MHC-I, latent viral reservoir, antiretroviral, Nef inhibitor

## Abstract

While antiretroviral drugs have transformed the lives of HIV-infected individuals, chronic treatment is required to prevent rebound from viral reservoir cells. People living with HIV also are at higher risk for cardiovascular and neurocognitive complications, as well as cancer. Finding a cure for HIV-1 infection is therefore an essential goal of current AIDS research. This review is focused on the discovery of pharmacological inhibitors of the HIV-1 Nef accessory protein. Nef is well known to enhance HIV-1 infectivity and replication, and to promote immune escape of HIV-infected cells by preventing cell surface MHC-I display of HIV-1 antigens. Recent progress shows that Nef inhibitors not only suppress HIV-1 replication, but also restore sufficient MHC-I to the surface of infected cells to trigger a cytotoxic T lymphocyte response. Combining Nef inhibitors with latency reversal agents and therapeutic vaccines may provide a path to clearance of viral reservoirs.

## 1. Introduction

More than 37 million people worldwide continue to live with the human immunodeficiency virus (HIV) and the pace of progress for reducing new infections, increasing treatment access, and ending acquired immunodeficiency syndrome (AIDS) deaths is unfortunately decelerating (UNAIDS 2020 Fact Sheet). Although combination antiretroviral therapy (cART) effectively prevents the development of AIDS in HIV-infected individuals, current drugs do not eliminate persistent viral reservoirs. Furthermore, the combination of residual HIV-1 gene expression and the toxicity associated with chronic cART drug exposure enhances the risk for cardiovascular disease, neuropathology, cancer, and premature death [1]. Thus, finding a cure for HIV-1 infection is of paramount importance to global health.

HIV-1 is an integrating lentivirus that directly targets the adaptive immune system, making the development of curative strategies very challenging. HIV-1 vaccine trials have thus far been largely unsuccessful, with just one vaccine candidate producing partial protection [2]. Considerable research efforts have also addressed therapeutic elimination of persistent HIV-1 reservoir cells. One approach, often referred to as ‘shock and kill’, involves latency reversal agents (LRAs) to reactivate the integrated provirus. In theory, re-expression of viral proteins may promote immune system recognition and killing of HIV reservoir cells [3]. Promising LRAs have been shown to induce latency reversal in mouse and non-human primate models of HIV infection in the presence of antiretroviral drugs. Recent examples include IL-15 receptor super-agonists [4] as well as a small molecule activator of non-canonical NF-κB signaling [5]. While these and other agents successfully induce HIV-1 expression in the presence of cART, neither treatment alone reduced the viral reservoir. Additional modalities are likely required to trigger an effective adaptive immune response targeting reactivated HIV-1 reservoir cells. In this review, we will focus on the development of inhibitors of the HIV-1 Nef accessory protein because of its important role in immune escape of HIV-infected cells. Addition of Nef inhibitors to a regimen of LRAs and therapeutic vaccines may enhance reduction of the latent reservoir to effect a functional cure.

## 2. HIV-1 Nef as a Rational Target for Antiretroviral Drug Development

Nef is a relatively small protein (27–30 kDa, depending on the isolate) that is packaged in the virion and is also expressed at high levels early in the viral life cycle. Structurally, Nef is composed of an N-terminal anchor region followed by a folded core. The anchor region is approximately 60 amino acids in length and is largely unstructured. A signal sequence for N-myristoylation plus a short amphipathic helix [6] in the anchor localize Nef to host cell membranes [7]. The anchor region is flexible, allowing the folded core to move off the membrane [8,9], accommodating interactions with host cell partner proteins. Because many protein partners for Nef are also membrane localized, mutants of Nef that cannot be myristoylated are often non-functional.

Nef lacks any known biochemical or enzymatic activities, functioning instead through interactions with a diverse range of host cell proteins at the plasma membrane, trans-Golgi network and other membranous sites. These interactions account for many Nef activities, including downregulation of cell-surface major histocompatibility complexes (MHC-I/II) and viral receptors (CD4/CXCR4/CCR5) [10,11], counteracting host cell restriction factors (e.g., SERINC proteins) [12,13,14], actin cytoskeleton remodeling [15], and stimulation of host cell signaling pathways (e.g., Tec- and Src-family kinases, [16]). Through these mechanisms, Nef allows HIV-infected cells to avoid immune surveillance by the host, prevents viral superinfection, and enhances viral infectivity and spread.

Early studies in non-human primates provided strong evidence that Nef is required for SIV and HIV-1 pathogenesis and the development of AIDS. Infection of Rhesus macaques with Nef-defective SIV results in low viral loads and a substantial delay in the onset of disease [17]. These findings are consistent with reports of individuals infected with Nef-defective HIV [18,19,20] in which viral loads remain low or undetectable and CD4^+^ T cell counts remain stable for years in the absence of cART. More recent studies have demonstrated an essential role for Nef in HIV-1 infection and T cell loss in vivo using humanized immune system mice [20,21]. Infection of these animals with HIV-1 results in viremia and rapid depletion of CD4 T^+^ cells from both the blood and tissue compartments. In contrast, Nef-defective HIV-1 replicates poorly in these animals and does not cause CD4^+^ T cell loss. Together, these animal and patient data demonstrate a central role for Nef in HIV pathogenesis, providing a strong rationale for the development of small molecule Nef antagonists as a new approach to HIV/AIDS therapy. As described in more detail below, Nef inhibitors currently in preclinical development not only suppress HIV-1 replication but also restore the anti-HIV cytotoxic T lymphocyte (CTL) response, illustrating their promise as part of an effective HIV cure strategy.

## 3. Early Efforts in Nef Drug Discovery

As described above, all known Nef functions require protein–protein interactions with host cell proteins which are mediated by conserved amino acid motifs [22,23]. One of the first Nef motifs to be recognized is the PxxPxR consensus sequence which forms a polyproline type II helix involved in binding a subset of Src-family kinase SH3 domains [24]. Subsequent X-ray crystallography of a Nef:SH3 domain protein complex revealed that in addition to the PxxPxR motif, SH3 domain binding also requires a hydrophobic pocket in the Nef core region that engages the RT-loop of the SH3 domain [25]. This hydrophobic pocket is formed by several conserved Nef residues, suggesting a possible target site for small molecule inhibitor binding. The conserved arginine in the PxxPxR motif makes a stabilizing salt bridge with an aspartate in the RT-loop of the SH3 domain. Recent structural and functional analyses have revealed that lentiviral Nef proteins have evolved a so-called ‘R-clamp’ which refers to amino acids that buttress this arginine to reinforce the salt bridge and stabilize the complex [26].

Combining in silico screening with a cell-based protein–protein interaction assay, Betzi et al. identified a hit compound that blocked Nef:SH3 complex formation [27]. This compound, a phenoxyacetamido benzoic acid analog called D1, partially reversed Nef-mediated MHC-I downregulation without affecting cell-surface CD4. This pharmacological effect is consistent with studies implicating the Nef PxxPxR motif in MHC-I but not CD4 downregulation. Indeed, a subsequent X-ray crystal structure of Nef in complex with the cytoplasmic tail of MHC-1 and the µ1 subunit of the AP-1 trafficking adaptor revealed a direct role for the PxxPxR motif in complex formation [28].

**Figure 1:**
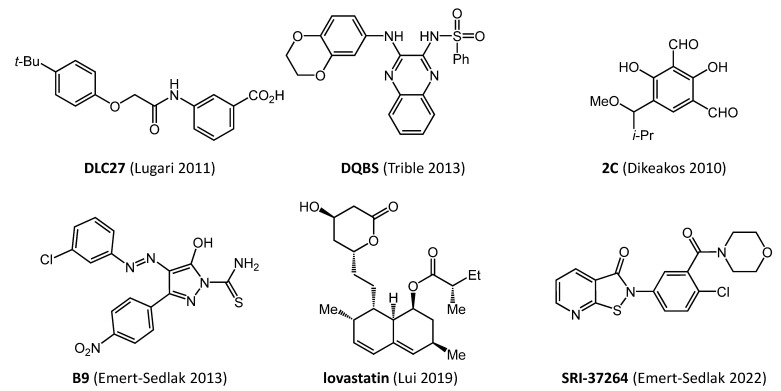
Chemical structures of direct binding HIV-1 Nef inhibitors. Initial studies describing the discovery of these compounds are referenced in parentheses. A comparison of their antiretroviral activities is presented in Table 1 [29,31,32,38,40].

Medicinal chemistry optimization led to an analog (DLC27) shown to bind directly to Nef by NMR with a *K_D_* value of about 1.0 μM (Structures of direct Nef inhibitors described in this review are shown in Figure 1; a comparison of their antiretroviral activities is summarized in Table 1). Chemical shift perturbations induced by DLC27 map to a hydrophobic pocket that includes conserved Nef residues Trp113 and Phe90 (numbering based on Nef crystal structure in complex with a Src-family kinase SH3 domain; PDB ID: 1EFN). Computational docking studies of DLC27 enabled development of additional analogs that interacted with a groove on the Nef surface [29]. One of these analogs sensitized Nef to proteolysis by the HIV-1 protease, suggesting that this compound may destabilize the structure of the Nef core. Unfavorable solubility properties and cytotoxicity prevented further assessment in viral infectivity and replication assays. Additional details of the discovery of early Nef inhibitors have been reviewed recently by Lurie, et al. [30], including docking models of predicted binding sites for these and other compounds on Nef crystal structures.

**Table 1:** Properties of HIV-1 Nef inhibitors. Compounds are listed in the order in which they are discussed in the main text (*nd*, feature not determined).

Compound	Direct Nef Binding	Inhibits HIV-1 Infectivity	Inhibits HIV-1 Replication	Restores Cell-Surface MHC-I	Triggers Anti-HIV CTL Response	Ref.
**DLC-27**	yes	*nd*	*nd*	yes	*nd*	[27]
**2C**	yes	yes	*nd*	yes	*nd*	[31]
**lovastatin**	yes	yes	*nd*	yes	yes	[32]
**concanamycin A**	no	*nd*	*nd*	yes	yes	[33]
**DFP analogs**	no	yes	yes	*nd*	*nd*	[34]
**B9**	yes	yes	yes	yes	yes	[35,36]
**non-azo B9**	yes	yes	yes	yes	yes	[35,36,37]
**SRI-37264**	yes	yes	yes	yes	*nd*	[38]
**FC-7976**	yes	yes	yes	yes	*nd*	[39]
**DQBS**	yes	yes	yes	yes	*nd*	[40]

## 4. Natural Products and Repurposed Drugs Inhibit Nef-Dependent Downregulation of MHC-I

Nef-mediated downregulation of MHC-I from the cell surface allows HIV-infected cells to escape immune surveillance [10]. This Nef function has been implicated in HIV persistence [41] and correlates with reservoir size in patient samples [42]. Discovery and development of inhibitors that reverse this Nef effect have promise in enhancing the adaptive immune response to clear HIV-infected cells.

MHC-I downregulation by Nef is a complex process that involves at least two discrete mechanisms. The first mechanism, referred to as the ‘signaling mode’ because it occurs early in the viral infection cycle, involves Nef-dependent activation of a Src-family kinase (Hck in macrophages and Lyn in T cells). This activation event occurs in the trans-Golgi network, where Nef is recruited by the phosphofurin acidic cluster 2 (PACS-2) adaptor protein [43]. Multiple studies have shown that Nef, through its PxxPxR motif, engages Src-family kinase SH3 domains as described above [16]. This results in displacement of the SH3 domain from its regulatory position on the back of the kinase domain, leading to constitutive kinase activation both in vitro and in cells (see Staudt et al. [16] for a recent review). Src-family kinase activity, in cooperation with ZAP-70/Syk kinases, ultimately triggers activation of PI3K, enhancing membrane levels of PIP_3_ and activation of Arf1 and Arf6. These small GTPases then induce retention of MHC-I through the AP-1 endocytic pathway [31,44]. Internalized MHC-I is trapped in vesicles in complex with Nef and AP-1 and prevented from recycling back to the plasma membrane. In a second mechanism, known as the ‘stoichiometric mode’, Nef associates directly with AP-1 and Arf1, trapping MHC-I molecules in the Golgi and preventing trafficking to the cell surface [45]. Both mechanisms involve coordinated association of Nef with AP-1 and the MHC-I cytoplasmic tail [28]. Endosomal trafficking pathways regulating MHC-I downregulation by Nef are the subject of other reviews [10,46].

Based on the signaling mechanism of MHC-I downregulation described above, small molecules that block association of Nef with Src-family kinases may prevent cell surface display of MHC-I in complex with HIV antigens. A synthetic analog known as ‘**2C**’ (Figure 1) which was derived from the *Streptomyces* metabolite UCS15A [47,48] works by this mechanism. **2C** inhibited Nef-dependent activation of the myeloid Src-family member Hck in vitro and prevent interaction of Nef with Lyn and Src, two other Src-family members susceptible to activation by Nef [49]. NMR studies showed interaction of **2C** with the Nef SH3 binding site and more strongly with a cleft between the β-sheet and the C-terminal α-helices of the folded Nef core. Thus, **2C** may prevent Nef-mediated Src-family kinase activation through allosteric inhibition of kinase recruitment. Nef interaction with Src-family kinases was blocked by **2C** in cell-based assays and it also repressed downregulation of MHC-I in HIV-infected cells [31]. As expected, **2C** had no effect on HIV-1-induced CD4 downregulation, which does not involve Src-family kinases and relies on the AP-2 endocytic adaptor [31]. In addition to rescue of cell-surface MHC-I, **2C** was also shown to block HIV infectivity in a Nef-dependent manner [50]. These proof-of-concept studies were among the first to establish that inhibition of Nef-dependent kinase signaling is a viable approach to the development of Nef antagonists with the potential to reverse MHC-I display and immune escape.

More recently, Liu et al. screened a library of FDA-approved drugs for inhibitors of Nef-mediated MHC-I downregulation by flow cytometry [32]. This approach identified lovastatin, a widely prescribed statin drug, as an antagonist of Nef-mediated downregulation of MHC-I. Lovastatin was also shown to enhance autologous CTL activity against HIV-infected cells and suppress viral rebound in CD4 T cells from HIV-infected individuals on cART. Lovastatin was shown to interact directly with Nef in vitro by surface plasmon resonance (SPR), albeit with K_D_ values in the 2-digit micromolar range. Lovastatin disrupted the interaction of Nef with the µ1 subunit of AP-1 in vitro in a co-immunoprecipitation assay, suggestive of a cellular mechanism of action. Lovastatin also inhibited Nef-mediated downregulation of both CD4 and SERINC5 at a concentration of 5 µM. These Nef target proteins are internalized via the AP-2 endocytic pathway, suggesting that lovastatin may be a good starting point for medicinal chemistry optimization of a general inhibitor of Nef-mediated receptor downregulation events. While these in vitro studies support repurposing of lovastatin for Nef inhibitor development, a previous clinical trial showed that lovastatin treatment had no effect on HIV-1 plasma viral loads or circulating CD4^+^ T cell counts in cART-naïve HIV-infected individuals [51]. This lack of activity may reflect the dose of lovastatin tested (40 mg once daily) which may not produce plasma levels sufficient to target the Nef protein based on the SPR data.

Another recent study reported that concanamycin A rescued MHC-I to the surface primary cells expressing Nef [33]. Concanamycin A is a member of the plecomacrolide family of natural products and has a complex 18-member ring structure. The effect of concanamycin A on Nef-mediated MHC-I downregulation was observed at sub-nanomolar concentrations and was specific to MHC-I without effects on CD4. Consistent with this observation, cells treated with the inhibitor showed reduced interaction of Nef with MHC-I and AP-1 by co-immunoprecipitation. Concanamycin A was active against Nef alleles from divergent HIV-1 subtypes as well as SIV and was effective against diverse MHC-I haplotypes. Concanamycin A-mediated rescue of MHC-I to the surface of HIV-infected cells enhanced the CTL response. The broad spectrum of activity of this compound suggests that it works by interfering with the host cell endocytic trafficking machinery, rather than by direct engagement of Nef itself. Nevertheless, this study provides additional support for the development of Nef antagonists that reverse MHC-I downregulation as part of a strategy to boost the host immune response to clear HIV-infected cells.

## 5. Coupling Nef to Src-Family Kinase Activation Enables Discovery of Direct Nef Antagonists

Nef lacks intrinsic biochemical or enzymatic functions, complicating the development of direct assays to screen for small molecule inhibitors. One successful approach to this problem has involved coupling of Nef to activation of the Src-family kinase Hck, a well-characterized Nef binding partner and host cell effector protein [16]. Hck is expressed in macrophages and other HIV target cells [52], and Nef-mediated Hck activation drives multiple Nef functions including replication enhancement and MHC-I downregulation as described above [24,31,43,44,53].

Hck activity is suppressed by intramolecular interaction of its SH3 domain with the linker connecting the SH2 and kinase domains. This interaction, together with binding of the SH2 domain to the tyrosine-phosphorylated C-terminal tail, allosterically stabilize the inactive conformation of the kinase domain [54,55]. As described above, Nef binds to Hck through its SH3 domain, displacing this negative regulatory interaction and inducing constitutive kinase activation both in vitro [56] and in cells [57]. Using recombinant purified inactive Hck and HIV-1 Nef, Emert-Sedlak et al. established in vitro kinase assay conditions in which Hck activation is dependent upon Nef [34]. Using this assay to screen a kinase inhibitor-biased library, they identified a series of 4-amino-diphenylfuranopyrimidine (DFP) analogs that preferentially inhibited Hck in the presence of Nef. While these inhibitors were presumed to act via the ATP-binding site in the Hck kinase domain, preferential inhibition in the presence of Nef suggested allosteric influence of the viral protein on the active site to favor compound binding. Additional evidence for this idea was provided by subsequent biophysical studies using hydrogen deuterium exchange mass spectrometry (HDX-MS) and the same recombinant Hck and Nef proteins [58]. In this study, Nef induced remarkably subtle changes in deuterium uptake by Hck, with the most significant occurring in the N-lobe of the kinase domain adjacent to the docking site for Nef on the SH3 domain. No changes in hydrogen exchange were observed in the Hck SH2 domain or C-terminal tail, indicating that this regulatory interaction is unaffected by Nef binding and consistent with earlier cell-based studies [59]. When HDX-MS was performed in the presence of a DFP-based inhibitor, the effect of Nef on Hck N-lobe dynamics was completely reversed [58]. These results show that constitutive Hck activation by Nef involves only modest changes to the conformational dynamics of the overall kinase structure that are reversed by DFP analogs.

DFP-based inhibitors blocked Nef-dependent enhancement of HIV-1 replication in U87MG cells with single-digit micromolar potency [34]. Inhibitors in this class were subsequently shown to block HIV-1 replication enhancement by Nef variants representative of all major HIV-1 subtypes [60]. Inhibition of viral replication correlated with suppression of endogenous Src-family kinase activity in the HIV-infected cells [60]. These studies show that Src-family kinase activation is a conserved feature of HIV-1 Nef alleles, and that activation of this kinase pathway is important for efficient HIV-1 replication.

In subsequent work, the Nef-Hck screening assay was adapted to full automation and used to screen a diverse chemical library of more than 220,000 compounds [35]. Initial hit compounds from the primary screen were then evaluated in dose–response studies using the Nef-Hck complex vs. Hck alone to identify Nef-dependent inhibitors. This approach identified about 60 compounds with at least a three-fold inhibitory preference for the Nef-Hck complex over Hck alone. Each compound was then screened for inhibition of Nef-dependent HIV-1 infectivity and replication in two different cell lines, identifying six compounds with Nef-dependent antiretroviral activity.

The most promising hit compound identified in the large-scale Nef-Hck screen was a diphenylpyrazolodiazene, termed ‘**B9′** for short (Figure 1). This compound displayed a 10-fold preference for inhibition of the Nef-Hck complex vs. Hck alone in the in vitro kinase assay. **B9** inhibited wild-type HIV-1 replication with an IC_50_ value in the three-digit nanomolar range in two different cell lines but did not affect replication of Nef-defective HIV-1, supporting a Nef-dependent mechanism of action. **B9** inhibited HIV-1 replication enhancement by a wide range of HIV-1 Nef subtypes which correlated with inhibition of endogenous Src-family kinase activation by Nef. **B9** also suppressed HIV-1 and SIV infectivity in the TZM-bl reporter cell line in a Nef-dependent manner [35]. Importantly, **B9** was also shown to interact directly with recombinant Nef by SPR, with a K_D_ value in the single-digit micromolar range.

**Figure 2:**
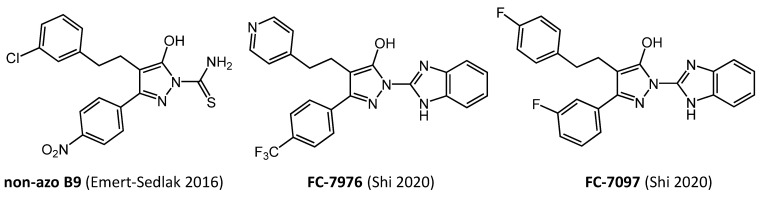
Medicinal chemistry optimization of **B9**. First-generation **B9** analogs replaced the diazene linker with one- or two-carbon bonds; a non-azo analog with a 2-carbon linker is shown. These analogs retained Nef binding and antiretroviral activity and demonstrated oral bioavailability [37]. **B9** and these non-azo analogs also restored sufficient MHC-I surface expression in primary CD4+ T cells to trigger a CTL response in vitro [36]. Subsequent analogs maintained the hydroxypyrazole core, which is essential for Nef interaction and antiretroviral activity, while diversifying substituents around the core as exemplified by analog **FC-7976**. This analog bound recombinant Nef by SPR with a K_D_ value in the 0.1 nM range and inhibited HIV-1 replication in donor PBMCs with an IC_50_ value of 0.7 µM [39]. Also shown is a related benzimidazole analog, **FC-7097**, which disrupted Nef homodimer formation by X-ray crystallography. See main text for details.

While **B9** interacted directly with Nef and displayed favorable antiretroviral properties, the presence of the diazene linker and nitro group represent medicinal chemistry liabilities. To begin to address this issue, a series of analogs were synthesized that replaced the diazene with one- and two-carbon linkers [37] (Figure 2). These compounds retained the original antiretroviral properties of **B9**, while also improving oral bioavailability and plasma half-life in mice. In subsequent work, **B9**, as well as these first-generation non-azo analogs, were found to restore MHC-I to the surface of HIV-infected CD4 T cells from a large cohort of HIV^+^ individuals. Moreover, when inhibitor-treated cells were co-cultured with autologous CD8 T cells expanded in the presence of HIV-1 antigenic peptides, the CD8 T cells became activated and displayed CTL responses against the infected CD4 target cells [36]. This result demonstrates that Nef inhibitors have the potential to enhance CTL-mediated responses to clear the latent viral reservoir.

A subsequent study adapted the same Nef-mediated Hck activation assay to a 1536-well format and screened more than 730,000 discrete compounds for additional Nef inhibitor leads [38]. Six unique hit compounds were identified that bound directly to recombinant Nef by SPR in vitro and inhibited HIV-1 replication in primary macrophages in the 0.04 to 5 μM range. Macrophages were used for initial assessment of antiretroviral activity because Hck is highly expressed in this HIV-1 host cell type, is directly activated by Nef as described above, and promotes viral replication [53]. An isothiazolone scaffold from this series was the starting point for synthesis of 84 additional analogs, many of which bound to recombinant Nef by SPR and inhibited HIV-1 infectivity in the low to submicromolar range. An isothiazolopyridinone analog from this series (**SRI-37264**; Figure 1) restored MHC-I expression to the surface of HIV-infected primary cells and disrupted a recombinant protein complex of Nef, the C-terminal tail of MHC-I and the μ1 subunit of the AP-1. Work with these compounds is consistent with earlier studies implicating Nef-mediated Hck activation as an early step in the MHC-I downregulation pathway described above. However, related isothiazolones are reactive electrophiles and interact with Cys proteases and other proteins with active site cysteines, which may limit their further development. Interaction with Nef, on the other hand, was reversible by SPR and did not result in stable adduct formation by mass spectrometry [38].

## 6. Hydroxypyrazole Nef inhibitor Analogs Display Potent Antiretroviral Activity in Primary Cells and Restore Cell-Surface MHC-I Expression

More recently, Shi et al. reported the synthesis of a diverse collection of more than 200 analogs of **B9** without the diazene functionality [39]. All analogs retained the hydroxypyrazole core present in **B9**, with a range of substituents present around the core (Figure 2). Interaction kinetics of each analog with Nef was assessed in vitro using SPR along with a cell-based assay for antiretroviral activity (TZM-bl reporter cell assay for infectivity described above). Introduction of a bulky benzimidazole or related substituent in place of the thioamide present in **B9** greatly enhanced Nef binding affinity in many cases. Binding and antiretroviral activity data for each compound were then integrated as a single ‘activity score’ which permitted ranking of overall analog efficacy. The top-scoring compounds bound to recombinant Nef by SPR with K_D_ values in the 0.1 to 10 nM range, with complete suppression of viral infectivity at 1.0 µM. The structure of one of the most active analogs, **FC-7976**, is shown in Figure 2.

The antiretroviral activity of the top-scoring hydroxypyrazole Nef inhibitor analogs were then assessed in HIV-infected peripheral blood mononuclear cells (PBMCs) from uninfected donors. PBMCs were infected with HIV-1 in the presence of each compound over a range of concentrations and cell viability was assessed in parallel. Five analogs inhibited Nef-dependent enhancement of HIV-1 replication in PBMCs in the single-digit nM range without cytotoxicity. In preliminary studies, **FC-7976** was also observed to suppress HIV-1 replication in HIV-infected human CD34^+^ hematopoietic stem cell-engrafted immunocompromised mice (S. Shu and T. Smithgall, unpublished results).

The most active Nef inhibitor analogs were also screened for rescue of Nef-mediated MHC-I downregulation using the CEM-SS cell line [39]. This T cell line is engineered to express the MHC class I allele HLA-A*02, and transfection of these cells with a Nef expression vector results in robust downregulation [61]. Use of this Nef-transfected cell line separates the effect of Nef on MHC-I from effects on viral infectivity and replication, thus simplifying interpretation of the results. The vector also carries a second expression cassette for GFP, which allows for gating of the transfected cell population by flow cytometry. All the hydroxypyrazole Nef inhibitor analogs restored cell-surface MHC-I expression to a greater extent than **B9** (10 to 25% rescue at 1.0 µM vs. 4% for **B9**). This observation is consistent with the enhanced potency of these compounds in the SPR, infectivity, and viral replication assays. Additional medicinal chemistry optimization is ongoing to improve the physiochemical and pharmacological properties of these inhibitors for in vivo assessment in animal models of persistent HIV infection.

## 7. Mechanism of Action of Hydroxypyrazole Nef Inhibitors—Clues from Structural Biology

Previous structural studies have established that HIV-1 Nef has a remarkable ability to form multiple homodimeric structures, despite a conserved fold of the core region [25,62]. Mutations that impact homodimer formation also disrupt most if not all Nef functions, including enhancement of infectivity, viral replication, host cell tyrosine kinase activation and cell-surface receptor downregulation (reviewed in Staudt et al. [16]). These observations suggested that hydroxypyrazole Nef inhibitors, which also disrupt multiple Nef functions, may do so by perturbing Nef homodimer structure and dynamics. Computational docking studies with the original Nef inhibitor **B9**, as well as first-generation non-azo analogs, suggested that these compounds recognize the Nef homodimerization interface, supporting this idea [35,37].

In preliminary structural studies, diffracting crystals of a recombinant Nef core protein bound to the Hck SH3 domain were produced in the presence of the representative hydroxypyrazole Nef inhibitor, **FC-7097** (J. Alvarado and T. Smithgall, unpublished results; **FC-7097** structure shown in Figure 2). The presence of the inhibitor resulted in monomeric crystal complexes of Nef with the SH3 domain, as opposed to the 2:2 dimer complexes previously observed in the absence of bound ligand (e.g., PDB ID: 1EFN). While Nef:SH3 crystallized as a monomeric complex in the presence of the inhibitor, the interface between Nef and the SH3 domain was unchanged. This observation suggests that hydroxypyrazole inhibitors may prevent Nef homodimer formation under certain conditions, and is consistent with earlier work showing that the **B9** parent compound disrupts Nef homodimerization in a cell-based fluorescence complementation assay [35]. These structural findings are consistent with published observations that hydroxypyrazole Nef inhibitors like **FC-7097** inhibit Nef-dependent activation of Hck as well as the Tec-family kinase Itk in transfected cells [39]. Importantly, the inhibitors have no effect on the kinases alone. Thus, the inhibitors may interfere with Nef homodimerization at the cell membrane which in turn prevents Nef-mediated kinase dimerization as required for autophosphorylation and activation. Further work is needed to map the complete inhibitor binding pocket within Nef and to determine whether the effect of **FC-7097** on Nef:SH3 quaternary structure is a mechanism shared by other inhibitors in this class.

While the role for Nef homodimers in tyrosine kinase activation is supported by biochemical and structural data [16], X-ray crystal structures of Nef in complexes with MHC-I [28] and CD4 [63], as well as the respective AP-1 and AP-2 subunits responsible for their downregulation, reveal Nef in a monomeric state. This raises an important question as to whether disturbance of Nef homodimer formation by hydroxypyrazole Nef inhibitors is involved in the mechanism of cell-surface MHC-I rescue by these compounds. One possibility is that Nef homodimers play an early role in the downregulation pathway. This idea is supported by structural studies demonstrating that Src-family kinase engagement induces a Nef conformation that exposes a residue (Asp123) essential for both MHC-I and CD4 downregulation [64]. Release of Nef from the kinase may then result in the final receptor complexes captured in the crystal structures. Alternatively, the inhibitors may affect cell-surface MHC-I indirectly, by preventing the Src-family kinase activation step necessary for MHC-I downregulation via the signaling mode described above. It is also possible that inhibitor binding interferes with assembly of the Nef complex with MHC-I and AP-1, as shown recently for inhibitors of the isothiazolone class [38]. Additional structural and mechanistic studies are required to unravel this aspect of inhibitor action.

## 8. Alternative Approaches to Nef Drug Discovery

Several other assay systems have been reported for the identification of Nef inhibitors. Trible et al. developed a yeast-based phenotypic screen to identify small molecules that inhibit Nef-dependent Hck activation [40]. This screen is based on the previous observation that active Src-family kinases, including Hck, cause cell-cycle arrest in yeast [49]. Thus, small molecules that inhibit Nef-dependent kinase activation restore cell growth, providing a straightforward read-out for hit selection. Co-expression with Nef was first shown to induce constitutive activation of Hck in yeast by the same biochemical mechanism observed in mammalian cells. Nef-activated Hck induced growth arrest in yeast that was reversed with an ATP-site Src-family kinase inhibitor, providing a positive control for the assay. Subsequent screening of a library of 2,500 drug-like compounds identified a diaminoquinoxaline benzenesulfonamide analog (**DQBS**; Figure 1) that inhibited Nef-dependent enhancement of HIV-1 replication in U87MG cells with an IC_50_ value of 130 nM. DQBS also blocked MHC-I downregulation by Nef in the H9 T cell line. The mechanism involved disruption of the Nef-mediated assembly and activation of the multi-kinase complex responsible for the signaling mode of MHC-I downregulation described above. Importantly, control assays demonstrated that DQBS did not directly affect Hck or ZAP-70 kinase activities, supporting direct action through Nef. Docking studies also supported direct interaction of this compound with Nef which was confirmed in differential scanning fluorimetry assays. DQBS also inhibited the replication of HIV-1 NL4-3 chimeras expressing Nef alleles representative of all M-group HIV-1 clades with similar nanomolar potency but did not affect the replication of Nef-defective HIV-1.

A final assay approach screened for direct inhibitors of Nef homodimer formation [65], which has been linked to many Nef functions as described above. This cell-based assay is based on bimolecular fluorescence complementation (BiFC), which directly reports protein–protein interactions and their localization in transfected cells [66]. For this assay, Nef was fused to non-fluorescent, complementary fragments of a yellow-shifted variant of green fluorescent protein (YFP). The YFP fragments were fused to Nef through its C-terminal tail to maintain N-myristoylation and membrane localization. The two Nef BiFC fusion constructs were then co-expressed in 293T cells, where Nef homodimer formation resulted in juxtaposition of the YFP fragments and reconstitution of the fluorophore, producing bright green fluorescence at the cell membrane. To simplify the assay for automation, the two Nef-BiFC fusion proteins plus a monomeric red fluorescent protein (mRFP) reporter were expressed from a single vector, separated by picornavirus ‘2A’ ribosomal skipping sequences for co-translation of all three proteins. This approach allowed normalization of the Nef-BiFC signal for homodimerization to the mRFP signal for expression. The Nef-BiFC to mRFP ratios from cells expressing wild-type Nef versus a dimerization-defective Nef mutant were very clearly separated, with Z’ factors in the 0.6 to 0.7 range. A fully automated pilot screen of approximately 1600 diverse compounds identified 10 hit compounds that reproducibly blocked Nef dimerization in the low micromolar range. While this study did not include follow-up data for inhibition of Nef function, it illustrates a more direct approach to identify small molecule Nef binders that may represent leads for further development.

## 9. Summary and Future Directions

Many studies have implicated Nef as a key factor in HIV-1 pathogenesis, including establishment and maintenance of the persistent viral reservoir [41]. Multiple proof of concept studies summarized here support the continued development of pharmacological approaches to target Nef, for which no FDA approved drugs currently exist nor are in clinical trials to our knowledge. Nef represents a challenging drug target, because it lacks intrinsic biochemical activity and has no active site or obvious pocket for inhibitor binding. That said, genetic and pharmacological evidence suggest that manipulation of Nef quaternary structure may be one key to global inhibition of Nef function. Of the many known Nef functions, pharmacological suppression of Nef-mediated MHC-I downregulation may be the key to an effective CTL response and viral clearance. Development of direct Nef inhibitors with physiochemical and pharmacokinetic properties amenable to testing in animal models of HIV latency represents a key milestone going forward. A continuing challenge in the development process is the identification of assay platforms that report Nef functions predictive of in vivo activity. While Shi et al. addressed this issue through the combination of direct Nef binding by SPR and cell-based HIV-1 infectivity assays plus subsequent assessment of MHC-I rescue in vitro [39], identification of the most promising compounds for clinical trials will ultimately require in vivo evaluation of effects on viral reservoirs. In the end, Nef inhibitors may have a key role in combination with LRAs and therapeutic vaccines to reduce or eliminate HIV reservoirs.
